# Changes in public knowledge and perceptions about antibiotic use and resistance in Jordan: a cross-sectional eight-year comparative study

**DOI:** 10.1186/s12889-021-10723-x

**Published:** 2021-04-19

**Authors:** Suzanne Abdelmalek, Rowan AlEjielat, Walid Abu Rayyan, Nidal Qinna, Dana Darwish

**Affiliations:** Faculty of Pharmacy and Medical Sciences, University of Petra, Amman, Jordan

**Keywords:** Antibiotic, Resistance, Awareness, Beliefs, Knowledge, Perception, Comparison study

## Abstract

**Background:**

Resistance to antibiotics is a growing problem, worldwide and particularly in developing countries like Jordan. Raising public awareness on appropriate antibiotic use is crucial to combat this problem. The current study describes the change in public Knowledge and attitudes towards the use of antibiotics over a period of 8 years.

**Methods:**

Two cross-sectional studies were performed 8 years apart on Jordanians of different age groups, and social settings, residing in Amman, Jordan. Convenience non-probability sampling techniques were used. In 2010, a questionnaire was distributed in paper form, whereas in 2018 snowball sampling was used to disseminate an identical electronic questionnaire. Chi-square test and post hoc analysis were done using the z-test to compare column proportions, adjustment for multiple testing using the Bonferroni method. Multiple logistic regression was used to adjust for case mix for each survey. Comparisons were made across the two studies and within each study.

**Results:**

A total of 711 participants in 2010 and 436 participants in 2018 were surveyed. Over the 8-year period, there was a significant improvement in the beliefs regarding the use of antibiotics such as disagreeing to keeping left over antibiotics for later use from 57 to 70% (*p* < 0.05) and disagreeing to buying antibiotics without physicians’ consent increased from 80 to 89% (*P* value < 0.001). There was no significant change in the beliefs that support self-medication such as: using antibiotics from a friend (72 to 77%) buying antibiotics without a prescription (42 to 45%), and getting information about medication use from leaflet without referring to a health care professional (60 to 63%). There were some areas of confusion regarding antibiotic range of effectiveness, and origin of resistance. Agreement about antibiotic resistance being a problem in Jordan increased significantly from 44 to 60% (*p* < 0.001). In addition, there was a significant increase in the percentage of participants who said that they don’t request antibiotics from physicians (56 to 75% (*P* ≤ 0.001) and who said they would trust physicians’ decisions about the necessity of antibiotics (70 to 83% *P* < 0.05).

**Conclusion:**

Findings indicate the need for better suited, and more inclusive, public educational campaigns.

**Supplementary Information:**

The online version contains supplementary material available at 10.1186/s12889-021-10723-x.

## Background

Antibiotics have been used to treat infections since the early twentieth century [1]. However, excessive, and unnecessary use have resulted in the development of resistance towards their effect [2]. This problem is well known to scientists who work in the field, on the contrary, a considerable proportion of the public remain unaware of it [3, 4]. In developing countries, antimicrobial misuse and abuse are evident. Generally, most antimicrobials can be freely purchased from community pharmacies without a medical prescription. Patients quite often do not comply to the correct dose, omit doses, or combine antibiotics with herbs believing that it would improve their effect [5].

Antimicrobial resistance (AMR) is growing at an alarming rate, hence it mandates intervening at different levels to effectively combat it. Efforts should be geared at the community level as well as the professional bodies, involving political, medical, veterinary, agricultural, industrial or environmental societies [6]. The public plays an important role in the emergence, spread and control of bacterial resistance to antibiotics [7]. This was highlighted in the World Health Organization (WHO) Global Strategy for Containment of Antimicrobial Resistance in 2001 [8], where factors contributing to anti-microbial resistance were identified. These were patients’ misperceptions, self-medication, poor adherence to dosage regimens and advertising and promotion [8]. As a remedy, educational activities were recommended to raise Knowledge on the appropriate use of antimicrobials, the importance of prevention measures such as immunization, vector control, handwashing, food hygiene, etc., and use of suitable alternatives to antimicrobials for the relief of symptoms. Furthermore, activating measures to encourage health care seeking behavior and prohibit self-initiation of treatment [8]. The WHO global action plan to tackle antimicrobial resistance (2015) listed several objectives to reach this aim. First, to take immediate measures to raise awareness of antimicrobial resistance and promote behavioral change, through public communication programs that target different audiences as well as consumers [9]. In Jordan, the Ministry of Health (MoH) along with WHO and academic institutes have been collaborating to raise public awareness on the proper use of antibiotics. Social media platforms have been widely leveraged for health promotion and launching of health awareness campaigns. Publishing awareness content on social media might allow international distribution and an indefinite exposure period [10]. Many studies assessed the behavior regarding antibiotic use in Jordan [11–17]. The current study, however, is a comparison report of Knowledge and perceptions about antibiotic use and resistance in Jordan across 8 years. It was conducted on a sample from the city of Amman (the capital of Jordan) in 2010 and another sample in 2018. We aimed at observing the change in public Knowledge and attitudes towards the use of antibiotics over the period of 8 years. We expected the public awareness towards antimicrobial resistance to improve as a consequence of the programs implemented by the government and WHO, which also coincided with the increased use of social media platforms over the past years.

## Methods

Two cross sectional studies were performed 8 years apart. The first study was conducted between July–August 2010, which involved using paper-based questionnaire. The second study was conducted between July – August 2018, involving an electronic survey. The questionnaires were identical and distributed to two unrelated populations.

The study included a sample of Jordanians residing in Amman, the capital city of Jordan. Convenience non-probability sampling was used. The paper-form questionnaire involved trained researchers who approached possible participants from multiple sites. The study sites included community pharmacy customers, coffee shops customers, students at universities etc. Participants’ consent was obtained prior to answering the questionnaire. Snowball sampling was used for the distribution of the electronic questionnaire in 2018. A link to the survey was sent to Facebook and WhatsApp groups, both professional and non-professional, initially, then each group was asked to send the questionnaire to other acquaintances regardless of education, gender, or occupation in sought of randomness. A brief description of the study and its aims was included in the beginning of the electronic survey and filling the questionnaire was considered as participant’s consent.

A sample size calculation based on a 95% confidence level, 5% margin of error and 0.5 estimated anticipated frequency in a population of around 1.5 million in Amman resulted in a minimum of 384 surveys to be collected for each year, however additional questionnaires were collected in both surveys to reduce bias.

### Design and development of questionnaire

Relevant literature was reviewed, and the questionnaire was compiled by two clinical pharmacists and a microbiologist. The questionnaire was designed in the Arabic language, to prevent any vagueness and was tested before administration to the public on a sample of 10 friends and family members. English version of the questionnaire is available as Additional file 3.

The questionnaire consisted of 22 questions. Three demographic questions. Thirteen questions in the form of statements for which the participants could choose an answer of either “I agree”, “I do not agree”, or “I do not know”. Three multiple choice questions, one “yes”, “no”, or “I don’t know” question, and two multi-answer questions (e.g. questions on indications of antibiotics and consequences of antimicrobial resistance).

The first part enquired about demographics, involving gender, age, and medical insurance. The second part asked about antibiotic use by participants (8 questions): ability to name an antibiotic, frequency of antibiotic use over the past 12 months, obtaining antibiotics from friends or relatives without consulting a physician, ability to obtain information on antibiotic use from drug leaflet versus a medical professional, keeping leftover antibiotics for future use, purchasing antibiotics without a prescription, completion of antibiotic regimen, and buying antibiotic against doctors’ recommendation. The third part also consisted of 8 questions. It asked about awareness of antibiotics action, indications for use, causes and consequences of antimicrobial resistance and the seriousness of the antimicrobial resistance problem in Jordan. In more detail, participants were asked if antibiotics work on bacteria or viruses, then they were presented with two sentences concerning the effect of antibiotics on common cold and were asked if they agreed or disagreed with the sentence or if they didn’t know the answer. They were also presented with a multiple answer question that contained possible indications for antibiotics. To assess Knowledge about resistance, participants were presented with two phrases that inquire about the origin of antibiotic resistance, a multi-answer question that asks about possible consequences of antibiotic resistance, and one phrase about the current status of antibiotic resistance in Jordan. The fourth and last part consisted of 3 questions concerned with the interaction between participants and physicians relevant to antibiotics use: requesting antibiotics from physicians, trusting the physicians’ decisions when not having antibiotics prescribed, and patients opinion about the physician who doesn’t prescribe antibiotics. Participants were asked to answer sincerely. The survey was anonymous, and no monetary compensation was given.

### Statistical analysis

Data were analyzed using the statistical software SPSS version 25. Descriptive analysis using frequencies and percentages was performed, as well as testing for statistically significant association between sample demographics and the level of awareness of antimicrobial resistance. Comparisons were done across the two studies and within each study. Statistical significance was determined using the chi-square test. Alternatively, Fisher exact test was used when criteria for chi-square test was not met. Post hoc analysis was done using the z-test to compare column proportions, adjustment for multiple testing using the Bonferroni method was also performed. A *P*-value of 0.05 was considered significant. Multinomial logistic regression was used to adjust for case mix for each survey, gender and insurance were included in the model as confounders. Outcome was reported as odds relative to the correct answer. Missing data were deleted from analysis as they were considered missing at random and expected not to introduce bias in the analysis. In 2010 The percent of missing data was less than 5% for all questions except for one question where it reached 7.6%. There were no missing data in 2018 since the questionnaire was submitted electronically.

## Results

### Demographics

A total of 711 participants in 2010 (50% males and 50% females) and 436 participants in 2018 (30.96% males and 69.04% females) were studied. Demographics are shown in Table 1.

**Table 1:** Demographics

Variable	2010	2018
**Gender**		
**Male**	356 (50.01%)	135 (30.96%)
**Female**	355 (49.9%)	301 (69.04%)
**Total**	711	436
**Age (years)**		
≤ 29	418 (58.79%)	164 (37.6%)
30–39	148 (20.82%)	104 (23.9%)
40–49	73 (10.27%)	84 (19.3%)
≥ 50	49 (6.89%)	84 (19.3%)
Missing	23(3.23%)	
**Total**	711	436
**Medical Insurance**		
**Yes**	428 (60.2%)	296 (67.9%)
**No**	275 (38.7%)	140 (32.1%)
**Missing**	8 (1.13%)	
**Total**	711	436

### Beliefs and attitudes towards antibiotic use

Eighty-three percent of our sample knew a name of an antibiotic in 2010 compared to 95.5% in 2018. One third (32.1%) of participants used antibiotics once in the previous year in 2010 compared to 55.5% in 2018 (*P* value < 0.05). In addition, participants who used antibiotics three times or more dropped significantly from 34.6% in 2010 to 20.1% in 2018 with no significant differences observed after adjusting for gender and insurance.

Table 2 summarizes participants’ attitudes towards antibiotic use in both studies stratified by gender and insurance status. Please refer to additional files (Table A, Table B) for full description of the analysis.

**Table 2:** Changes in beliefs and attitudes across the 8 year

	Total 2010 ^a^	Total 2018		Female 2010	Female 2018		Male 2010	Male 2018		Insured 2010	Insured 2018		Uninsured 2010	Uninsured 2018	
Belief/attitude	N(%)	N(%)	sig	N(%)	N(%)	sig	N(%)	N(%)	sig	N(%)	N(%)	sig	N(%)	N(%)	sig
**It is good to keep remaining antibiotic doses at home for later use upon need**															
Agree	251(35.1)	101(23.2)	*	79(22.32)	62(20.6)	NS	84 (23.9)	39 (28.9)	NS	139 (32.6)	62 (20.9)	*	107 (39.5)	39 (27.9)	*
**Do not agree**	**406(57.4)**	**303(69.5)**	*	**264(74.58)**	**221(73.4)**	**NS**	**247 (70.2)**	**82(60.7)**	*	**263 (61.6)**	**211 (71.3)**	*	**140 (51.7)**	**92 (65.7)**	*
Do not know	50(7.1)	32(7.3)	NS	11(3.11)	18(6.0)	NS	21(6.0)	14(10.4)	NS	25 (5.9)	23 (7.8)	NS	24 (8.9)	9 (6.4)	NS
**I don’t mind using antibiotics from a friend or a relative without consulting a doctor**															
Agree	163(23.1)	87 (20)	NS	79 (22.3)	55 (18.3)	NS	84 (23.9)	32 (23.7)	NS	98 (23.0)	60 (20.3)	NS	63 (23.2)	27 (19.3)	NS
**Do not agree**	**512(72.4)**	**337 (77.3)**	**NS**	**264 (74.6)**	**241 (80.1)**	**NS**	**247 (70.2)**	**96 (71.1)**	**NS**	**315 (73.8)**	**229 (77.4)**	**NS**	**190 (70.1)**	**108 (77.1)**	**NS**
Do not know	32(4.5)	12 (2.8)	NS	11 (3.1)	5 (1.7)	NS	21 (6.0)	7 (5.2)	NS	14 (3.3)	7 (2.4)	NS	18 (6.6)	5 (3.6)	NS
**I don’t mind buying an antibiotic from a pharmacy without a prescription**															
Agree	386 (54.6)	227 (52.1)	NS	301 (66.4)	160 (53.2)	*	135 (44.4)	67 (49.6)	NS	212 (49.6)	139 (47.0)	NS	166 (61.3)	88 (62.9)	NS
**Do not agree**	**299 (42.3)**	**198 (45.4)**	**NS**	**141 (31.1)**	**134 (44.5)**	*	**158 (52.0)**	**64 (47.4)**	**NS**	**203 (47.5)**	**148 (50.0)**	**NS**	**95 (35.1)**	**50 (35.7)**	**NS**
Do not know	22 (3.1)	11 (2.5)	NS	11 (2.4)	7 (2.3)		11 (3.6)	4 (3.0)	NS	12 (2.8)	9 (3.0)	NS	10 (3.7)	2 (1.4)	NS
**I know how to use the antibiotic through reading the accompanied leaflet without consulting with a doctor or a pharmacist**															
Agree	242 (34.4)	144 (33.0)	NS	114 (32.4)	105 (34.9)	NS	128 (36.6)	39 (28.9)	NS	151 (35.7)	99 (33.4)	NS	87 (32.1)	45 (32.1)	NS
**Do not agree**	**421 (59.9)**	**275 (63.1)**	**NS**	**217 (61.6)**	**187 (62.1)**	**NS**	**203 (58.0)**	**88 (65.2)**	**NS**	**255 (60.3)**	**185 (62.5)**	**NS**	**165 (60.9)**	**90 (64.3)**	**NS**
Do not know	40 (5.7)	17 (3.9)	NS	21 (6.0)	9 (3.0)	NS	19 (5.4)	8 (5.9)	NS	17 (4.0)	12 (4.1)	NS	19 (7.0)	5 (3.6)	NS
**I buy antibiotics even if the doctor is convinced that it is not needed for my case**															
Agree	89 (12.7)	34 (7.8)	*	32 (9.1)	21 (7.0)	NS	57 (16.3)	13 (9.6)	NS	44 (10.4)	29 (9.8)	NS	44 (16.3)	5 (3.6)	*
**Do not agree**	**562 (79.9)**	**387 (88.8)**	*	**294 (83.3)**	**271 (90.0)**	*	**267 (76.5)**	**116 (85.9)**	*	**353 (83.3)**	**259 (87.5)**	**NS**	**202 (74.8)**	**128 (91.4)**	*
Do not know	52 (7.4)	15 (3.4)	*	27 (7.6)	9 (3.0)	*	25 (7.2)	6 (4.4)	NS	27 (6.4)	8 (2.7)	*	24 (8.9)	7 (5.0)	NS
**You can stop taking your antibiotic when you start feeling better**															
Agree	168 (24.0)	66 (15.1)	*	78 (22.2)	38 (12.6)	*	90 (25.9)	28 (20.7)	NS	96 (22.7)	46 (15.5)	*	68 (25.3)	20 (14.3)	*
**Do not agree**	**488 (69.6)**	**364 (83.5)**	*	**251 (71.3)**	**261 (86.7)**	*	**236 (67.8)**	**103 (76.3)**	**NS**	**306 (72.3)**	**248 (83.8)**	*	**178 (66.2)**	**116 (82.9)**	*
Do not know	45 (6.4)	6 (1.4)	*	23 (6.5)	2 (0.7)	*	22 (6.3)	4 (3.0)	NS	21 (5.0)	2 (0.7)	*	23 (8.6)	4 (2.9)	*

Correct answer is bolded; NS is not significant; *P values < 0.05; ^a^ There were some missing answers in 2010

When participants were asked if they agreed with the statement “It is good to keep remaining antibiotic doses at home for later use upon need” there was a significant change in opinions between 2010 and 2018. The percent of participants (69.5%) who disagreed to the statement in 2018 significantly exceeded those in 2010 (57.4%). Females (73.4%) significantly exceeded males (60.7%) in their correct answer in 2018 (*P* < 0.05) but not in 2010 (*P* = 0.12). However, after adjusting for insurance status, males in 2010 were 1.43 times more likely than females to agree with the sentence, 95% CI (1.031–1.969), *p* = 0.032. And in 2018 males were 1.7 times more likely than females to agree with the sentence, 95% CI (1.041–2.694) *P* = 0.034. When adjusted for gender the insured were less likely to agree with the sentence in 2010 (odds 0.657, 95% CI (0.472–0.915), *P* = 0.013* but not in 2018.

When participants were asked whether they agreed with the statement “I don’t mind using antibiotics from a friend or a relative without consulting a doctor “the majority of participants in both years disagreed. The percentages were 72 and 77% for 2010 and 2018, respectively, with a 5% increase in people who disagreed across the 8 years. Females-positive responses (80.1%) significantly exceeded male-responses (71.1%) in 2018 (*P* < 0.05) but not in 2010 (*P* = 0.146). After correcting for insurance status, there were no differences between males and females on the “agree” answer. But males were 2.36, and 3.47 times more likely to answer “I do not know” in 2010 and 2018 respectively (*P* = 0.027, 0.038). Insured participants after correcting for gender were less likely to answer I do not know in 2010 (odds 0.414, 95% CI (0.199–0.861, *P* = 0.018) but not in 2018, 2010 were 1.43 times more likely than females to agree with the sentence, 95% CI (1.031–1.969), *p* = 0.032. And in 2018 males were 1.7 times more likely than females to agree with the sentence, 95% CI (1.041–2.694) *P* = 0.034. When adjusted for gender the insured were less likely to agree with the sentence in 2010 (odds 0.657, 95% CI (0.472–0.915), *P* = 0.013* but not in 2018.

No significant change was found across the years in opinions regarding buying antibiotics from a pharmacy without a prescription. Those who disagreed with the sentence regarding antibiotic purchase without a prescription constituted 42.3% in 2010 and 45.4% in 2018. However, a statistically significant difference was observed when responses were stratified by medical insurance status. In both years insured participants significantly disagreed to the statement compared to uninsured ones (49.6% vs 35.5% in 2010 (*P* < 0.05) and 50% vs 35.7% (*P* < 0.05) in 2018). Which remained significant after correcting for gender. Interestingly, female correct responses significantly (*P* < 0.05) increased over the years while males’ correct responses did not.

Over half of participants in both years disagreed with the statement “I know how to use the antibiotic through reading the accompanied leaflet without consulting with a doctor or a pharmacist”, 59.8% in 2010 compared to 63% in 2018 with no significant difference between responses in the 2 years, even after correcting for confounders.

The percentage of participants who disagreed with the statement “I buy antibiotics even if the doctor is convinced that it is not needed for my case” increased significantly from 79.9% in 2010 to 88.8% in 2018 (*P* value < 0.001). Females percentage (83.3%) significantly exceeded that of male percentage (76.5%) in 2010 (*P* < 0.05), but there was no significant difference between them in 2018. This remained significant after adjusting for insurance, males were 2.19 times more likely to agree in 2010 (95%CI 1.36–3.52, *P* = 0.001) but there was still no difference in 2018. A statistically significant (*P* < 0.05) difference in the answers to this question was observed between age groups in 2018 (92.7% of the age group ≤29 disagreed with the sentence, 90.4% of the age group 30–39, 85.7% of the age group 40–49, 82.1% of the age group ≥50). Having a medical insurance seemed to influence answers to this question. In 2010, 83.3% of people who were insured did not agree with the statement compared to 74% of those not insured. However, in 2018, 87.5% of people who were insured did not agree with the statement, compared to 91.4% of people who were not insured. These differences were significant in both years after correcting for gender. Insured people were less likely to agree to the sentence, i.e. they didn’t buy antibiotic if the Dr. didn’t think it is needed in 2010, (odds 0.0507, 95% CI (0.319–0.805), *P* = 0.004) but in 2018 insured participants were 3 times more likely to agree to the sentence (odds 2.918, 95% CI (1.102–7.729), *P* = 0.031).

The percentage of people who disagreed with the statement“ you can stop taking your antibiotic when you start feeling better” increased significantly (*P* < 0.001) from 70% in 2010 to 83% in 2018. Females’ correct response (86.7%) exceeded males’ correct responses (76.3%) significantly (*P* < 0.05) in 2018 but not in 2010.

### Knowledge about antibiotics effects and causes of resistance

A. ***Knowledge about antibiotic range of effectiveness and indications.***

**Fig. 1:**
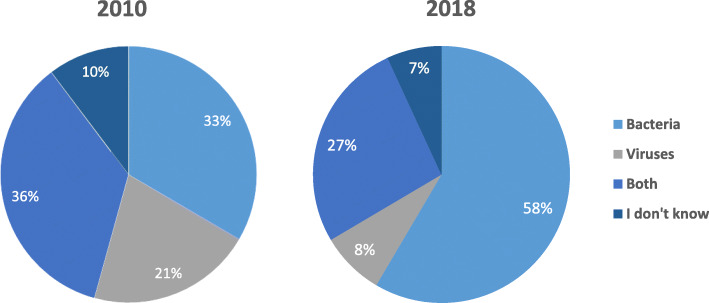
Change in Knowledge about range of antibiotics effectiveness. **a**: 2010, **b**: 2018. *P* values * < 0.05. < 0.001**

The percent of people who knew that antibiotics are effective against bacteria increased significantly from 33% in 2010 to 58% in 2018 (*p* < 0.001). Females 37% significantly answered more correctly compared to males 29.9% in 2010 (*P* value < 0.05), but there was no difference between them in 2018 (57.8% vs 58.8% for males and females respectively). The percent of people who stated that it is effective against viruses also decreased significantly from 21% in 2010 to 8% in 2018 (*p* < 0.001) (Fig. 1a, b). A substantial percentage of participants (35.4% in 2010 and 27% in 2018) thought that they can be used to treat both bacteria and viruses.

**Fig. 2:**
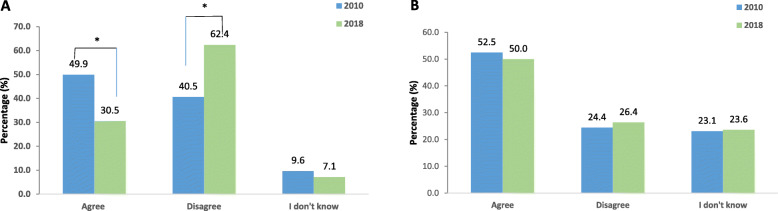
Changes in the Knowledge about antibiotics use. The following statements were presented to participants: **a**. Antibiotics accelerate recovery from common cold. **b**. “Runny nose accompanied by a colored discharge requires antibiotic therapy”. *P* values * < 0.05. < 0.001**

“Do antibiotics accelerate recovery from common cold?” Fig. 2a, shows that there was a significant (*p* < 0.01) increase in the percentage of participants who disagreed with this statement in 2018. There was a significant difference in answers to this question between age groups in 2018 (*P* < 0.05). 51.2% of the age group ≤29 disagreed to the statement 70.2% of the age group 30–39, 66.7% of the age group 40–49, 70.2% of the age group ≥50 disagreed. There was no effect for gender even after correcting for insurance.

There was no significant change over the years in the perception of participants regarding the relation between the color of nasal discharge and the necessity for use of antibiotics (Fig. 2b). However, there was a significant variation in the percentage of correct answers between age groups in 2018 (*P* < 0.05). 20.1% of the age group ≤29 disagreed with the statement: “Runny nose accompanied by a colored discharge requires antibiotic therapy”, 33.7% of the age group 30–39, 28.6% of the age group 40–49, 27.4% of the age group ≥50. There was no effect for gender nor insurance even after correction.

**Fig. 3:**
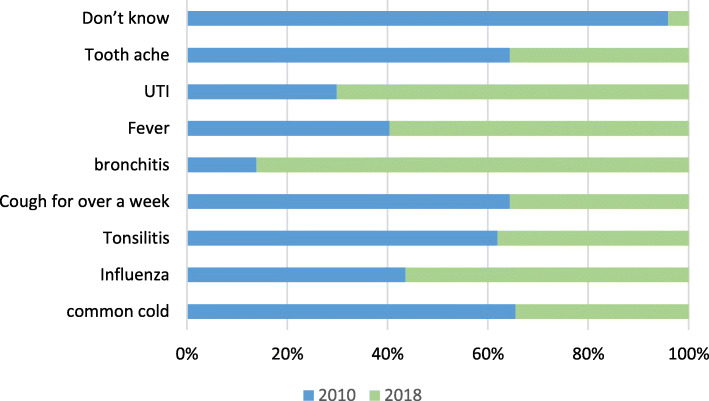
Perceptions about indications for antibiotics use

Knowledge of participants about the indications of antibiotics for treating different ailments has changed significantly. The percent of people who answered that antibiotics can be used to treat common cold, toothache and gum disease, cough longer than a week, or throat pain decreased. The percent of people who answered that antibiotics can be used to treat UTI, fever, and bronchitis increased. The percent of people who answered “I do not know” decreased significantly, and lastly there was no significant change in people’s opinions regarding the use of antibiotics in treating influenza (Fig. 3).

B. ***Knowledge about antibiotic resistance***

#### Origin of antibiotic resistance

**Fig. 4:**
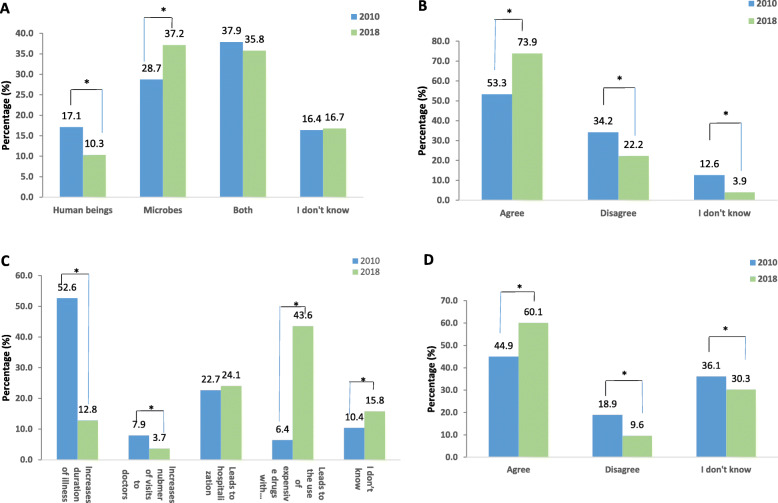
Knowledge about antibiotic resistance. **a** Origin of antibiotic resistance. **b**. Excessive use of antibiotics increase the chance of creating resistance. **c** Consequences of antibiotic resistance. **d** Antibiotic resistance is a problem in Jordan. *P* values * < 0.05. < 0.001**

The percentage of participants who answered that microbes are the source of antibiotic resistance increased from 28.7% in 2010 to 37.2% in 2018 (*p* = 0.002) and the percentage of people who answered that the source was “human beings” decreased from 17.1% in 2010 to 10.3% in 2018 (*p* = 0.002). The percentage of people who did not know remained almost the same, and a considerable percentage of participants thought that resistance is formed by both microbes and man: 37.9% in 2010 and 35.8% in 2018 (Fig. 4a). Responses were not age nor gender nor insurance influenced.

“Excessive use of antibiotics increases the chance of antibiotic resistance” There was a significant increase (*p* < 0.001) in the percentage of participants who agreed with the above statement from 53.5% in 2010 to 73.9% in 2018. (Fig. 4b). There was no effect for gender nor insurance even after correction.

#### Consequences of resistance

When we asked about the consequences of antibiotic resistance, we had varying results (Fig. 4c). There was a significant decrease in answers to the statement “It leads to an increase in the duration of illness” and a significant increase in answers to “It leads to the use of more expensive drugs with more side effects”. Which was also not influenced by age or gender.

#### Current status of resistance in Jordan

60.1% of the sample in 2018 agreed that antibiotic resistance is a problem in Jordan opposite to 44% in 2010 (*p* < 0.001) (Fig. 4d). There was no influence for gender or insurance even after correction.

### Patient -physician relationship

**Fig. 5:**
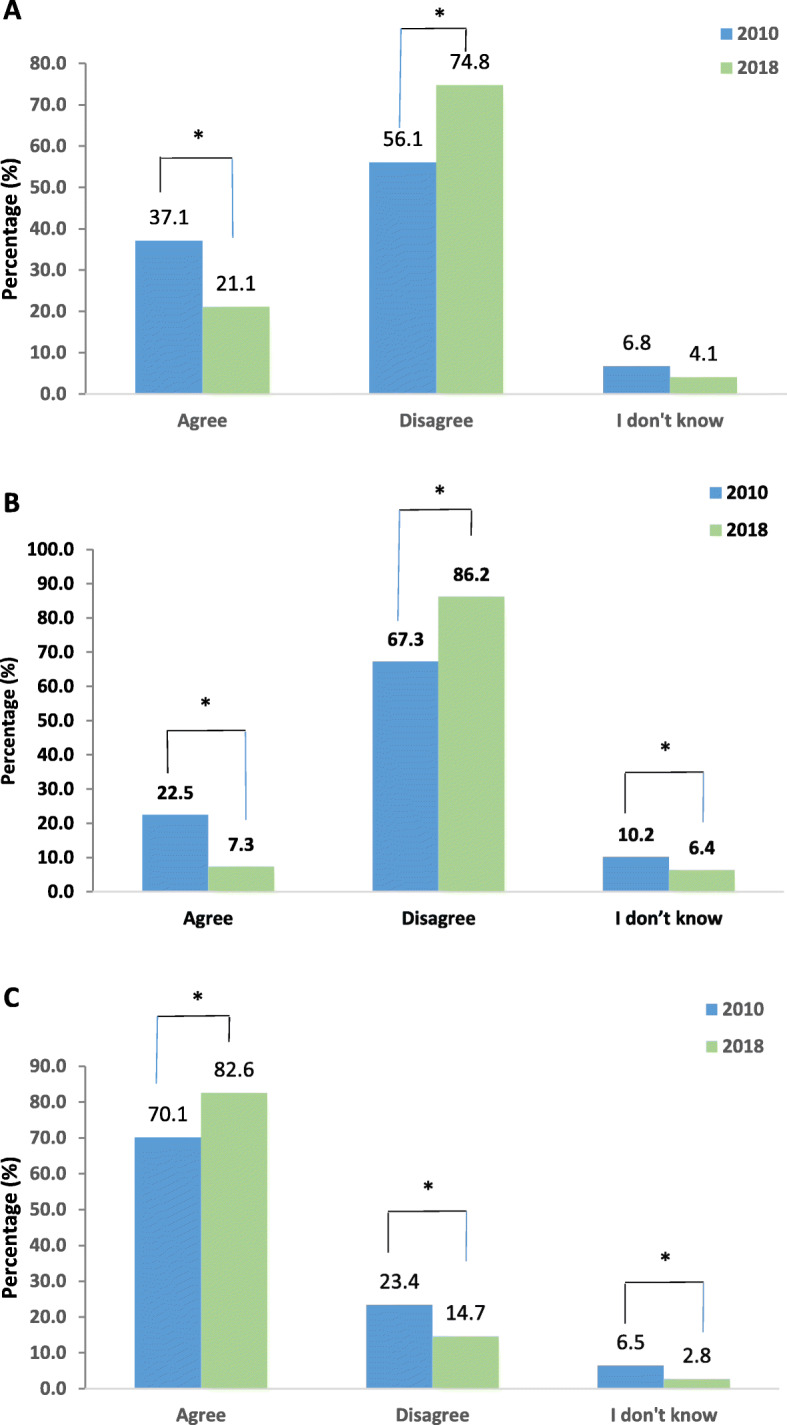
Patient -Physician relationship. The following statements were presented to participants. **a** I ask a doctor to prescribe an antibiotic for me if he does not prescribe one. **b** The Doctor who does not prescribe antibiotic, when the patient believe he should, is an incompetent doctor. **c** I trust the Doctor’s decision whether he/she prescribes an antibiotic or not. *P* values * < 0.05. < 0.001**

“I ask the doctor to prescribe an antibiotic for me if he doesn’t prescribe one”. There was a significant increase in responses which disagree with this statement from 56.1% in 2010 to 74.8% in 2018 (*P* ≤ 0.001) (Fig. 5a). Males were 1.45 times more likely to agree to the sentence in 2010 (95% CI 1.053–2.009, *P* = 0.023) but there was no effect of gender in 2018 after correcting for insurance status.

There was also a significant change in responses to the statement “The doctor who doesn’t prescribe antibiotic, when the patient believes he should, is an incompetent doctor.” The percent of people who disagreed increased significantly from 67.3 to 86.2% (*p* ≤ 0.05) (Fig. 5b). A significant (*P* < 0.05) difference across age groups in 2018 was observed: 80.5% of the age group ≤29 disagreed, 93.3% of the age group 30–39, 82.1% of the age group 40–49, and 92.9% of the age group ≥50. After correction, males were 1.48 times more likely to agree with the sentence in 2010 (95% CI 1.015–2.146, *P* = 0.042) but no significant differences were found in 2018.

There was a significant increase in percentage of people who agree with the statement “I trust the doctor’s decision whether he/she prescribes an antibiotic or not”. 70.1% answered “agree” in 2010 vs. 82.6% in 2018 (*P* < 0.05), (Fig. 5c). This indicates a better trusting relationship with physicians over the years. This was observed to be significantly age driven in 2018 with 75.6% of the age group ≤29 agreeing, 81.7% of the age group 30–39, 85.7% of the age group 40–49, and 94% of the age group ≥50 (*P* < 0.05). There was no influence for gender nor insurance even after correction.

## Discussion

Antibiotic resistance is a serious problem that is growing worldwide. To effectively combat antimicrobial resistance, one of the main objectives that must be achieved is to improve awareness and understanding of antimicrobial resistance through effective communication, education and training [9]. Many studies assessed public Knowledge and attitudes towards antibiotic use and resistance whether worldwide or in Jordan [11, 15, 17–21]. In the current study, comparison of public Knowledge over the years enabled characterization of the strengths and weaknesses in public awareness of antibiotic use and resistance over time. Variation in the case mix of participants in both surveys that was created with the use of different non-probability sampling techniques, was adjusted for by the use of the regression model The Snowballing technique that was used for the dissemination of the electronic questionnaire, may have resulted in sampling bias such as referral of the questionnaire to people who have similar traits or to those who utilized social media platforms used for the distribution of the questionnaire, overlooking those who choose not to use these platforms. On the other hand, possibility of bias also existed in the distribution of the paper form questionnaire by the trained researchers who selected participants at their convenience, where they approached people in their vicinity such as community pharmacy customers, coffee shops customers, students at universities etc. This also may have led to the exclusion of certain categories of people, such as those who are not able to reach mentioned places due to age, sickness or occupation. These sampling techniques may have created unavoidable underrepresentation of certain groups, which would require interpretation of the results with reservation. The findings showed improvement in awareness in some aspects and lack of change in others. The significant increase in disapproval of stopping antibiotics when symptoms improve is a positive change and conforms to another study performed in Northern Jordan, where 61% of participants believed the same [15]. On the other hand, public views in terms of purchasing antibiotics without a prescription did not change over time, which also agrees with previous studies, e.g., Shehadeh et al. (2012) in which [17] around one-third of respondents bought antibiotics directly from the pharmacy without a prescription [17]. This could be attributed to the unrestricted access to antibiotics from pharmacies, considering pharmacy a reliable source of information to advise on therapy, the tendency towards self-medication and relying on interpreting information in the drug leaflet to know about medication. It can also be explained by the patients’ financial and insurance status. Many of those who cannot afford going to the physician prefer to consult with the local pharmacist. Findings in the current study related to medical insurance further support this explanation. As expected, those uninsured would agree more to buying antibiotics without a prescription compared to those insured. In Jordan, public health care facilities provide all medicines free of charge for patients with health insurance. Moreover, for those with private insurance, medical coverage and the level of copayment depend on the specific insurance policy that has been purchased [22]. Thus, it would be feasible to get antibiotics through insurance, and thereby the need to buy it without a prescription will be reduced.

The number of people who used antibiotics once over the previous year increased significantly in 2018 compared to 2010. The multiple antibiotics use significantly decreased, which was not influenced by insurance status, age, or gender. This indicated a decrease in patients’ demand for antibiotics, particularly in conjunction with the evident change in beliefs on not requesting doctors to prescribe antibiotics or not perceiving doctors as incompetent if antibiotics were not prescribed.

Guidelines for the use of antibiotics for upper respiratory tract infections do not recommend using antibiotics unless bacterial involvement has been proven, and this requires a visit to a physician and a lab test [23]. The significant rise in the Knowledge of indications for antibiotics being bacterial and not viral infections is worth noting. Nonetheless, a notable proportion still thinks antibiotics are effective again both. In an earlier study by Shehada et al. [17] the authors also reported confusion regarding the range of effectiveness and use of antibiotics. Which, according to the results of the current study remained present.

The change in Knowledge about the causes and effects of antibiotics resistance was also positive. There seemed to be an increase in the Knowledge that antibiotic resistance originates in microbes; however, a considerable percentage of participants still thought that resistance originates in both microbes and man. Some misconceptions among individuals may be that since resistance starts in human beings, they would be protected if humans stop taking antibiotics. This and other misconceptions need to be emphasized and corrected in future educational campaigns.

Public awareness about the consequences of emerging antibiotic resistance have improved significantly. This was noticed primarily in the significant decrease in the percentage of participants who answered: “I don’t know”. Nevertheless, answers varied across the years. A direct reflection of the tightened economic status in Jordan could be seen in the change of participants’ responses to the perceived consequences of the spread of antibiotic resistance. There was a significant increase in the percentage of people who answered: “It leads to the use of more expensive drugs with more side effects”; 43.6% of the people in 2018 perceived this answer as a consequence compared to 6.4% in 2010. This change in belief could be a driving force for changing behaviors towards antibiotic use, especially when recognizing the presence of an antibiotic resistance problem in Jordan has improved significantly. Educational campaigns can undoubtedly use this information to design content to enhance public Knowledge, especially when about a third of our participants still reported that they do not know that antibiotic resistance is a problem in Jordan.

Patient pressure on physicians has always been considered a significant antibiotic misuse factor. Several studies have shown that physicians’ antibiotic prescribing patterns varied in response to patient pressure and expectations [24, 25]. In the current study, there was a significant increase in the percentage of participants who disagreed with the statement “I request the doctor to prescribe antibiotics if he didn’t already do it” and those who said they trusted the Doctor’s decision on the necessity of antibiotics. This response suggests an increased awareness and trust in the physician’s role in determining the need for antibiotics.

We expected the age group 20–40 to report more awareness, mainly due to the increased exposure to social media. On the contrary, we noticed higher attention in the older generation (aged 60 and above). Females were generally more open to change in perceptions about antibiotic use than men.

The advancement in telecommunications over the past few years has been evident. The use of social media and the Internet became more prevalent. According to NapoleonCat. Stats, since Facebook was introduced in Jordan in 2007 and until December 2018, 5,470,000 users were registered, which accounted for 52.6% of the entire population [26]. According to Statcounter Global Stats, the most popular social media platforms in Jordan are Facebook, YouTube, and Instagram in order [27]. Many YouTube videos on proper antibiotic use and antibiotic resistance have been published online from different sources [28–30], which were also circulated on Facebook, and others were broadcasted on television and radio and disseminated in newspapers. Moreover, social media activity on the same subject has been growing in the form of short informative videos, comics, and stories about treatment failures due to infections with antibiotic-resistant strains, proper use, and consequences of improper use. Together, these social media campaigns and the efforts put forth by the MoH, WHO and academia in Jordan could have improved public awareness on antibiotic use and resistance, as well as the physician-patient relationship.

### Limitations

The following limitations were encountered during the conduct of this comparative study. First, information on medical education was not collected in both studies. Even though the assumption could be made that people with health-related education would be more aware of proper antibiotic use, previous studies from Jordan showed otherwise [31].

We did not ask a specific question regarding the use of the Internet or the exposure to social media, which would hinder assessing the impact of social media campaigns.

The response rate in 2018 study could not be calculated because the tool was disseminated electronically. Only returned questionnaires were counted.

Although we attempted to correct the different participant characteristics in the two surveys by 3-way stratification and multinomial logistic regression, we cannot assume that this sample represents the population residing in Amman. Due to these limitations, the results of this study need to be interpreted with caution.

## Conclusion

These findings indicated the need for more adequate public education campaigns. These campaigns should target different age groups and multi-faceted modalities. Despite the improvement in awareness, some confusion still existed in antibiotic use and resistance. Emphasis should be laid on providing Arabic content (as Arabic is the mother tongue of most of the population), publicizing the medical websites that target non-medical people, and creating infographics that have proven to be an easy and effective way to present information [32]. Lastly, pharmacists’ role in spreading awareness and education must be highlighted since they are the first point of contact by the public to purchase their antibiotics without a prescription.

## Supplementary Information

**Additional file 1: Table A.** Chi Square test results comparing differences across the years stratified by gender and insurance.

**Additional file 2: Table B.** Multinomial logistic regression controlling for gender and insurance as confounders in the surveys case-mix. Odds presented as the odds of the wrong answer relative to the right answer for each possible option.

**Additional file 3.** Questionnaire in English version.

## Data Availability

The datasets used and/or analyzed during the current study are available from the corresponding author on reasonable request.
