# Supplementary material for: Changes in public knowledge and perceptions about antibiotic use and resistance in Jordan: a cross-sectional eight-year comparative study
Source: BMC Public Health. 2021 Apr 19;21:750. doi: 10.1186/s12889-021-10723-x (PMC8054398; doi:10.1186/s12889-021-10723-x)
Supplement: Supplementary file 1 — Additional file 1: Table A. Chi Square test results comparing differences across the years stratified by gender and insurance. [file 12889_2021_10723_MOESM1_ESM.docx]

Table A

Chi Square test results comparing differences across the years stratified by gender and insurance.

|  | Total  2010 # | Total  2018 |  | Female  2010 | Female  2018 |  | Male  2010 | Male  2018 |  | Insured  2010 | Insured  2018 |  | Uninsured  2010 | Uninsured  2018 |  |
| --- | --- | --- | --- | --- | --- | --- | --- | --- | --- | --- | --- | --- | --- | --- | --- |
| **Belief/attitude** | N (%) | N (%) | sig | N (%) | N (%) | sig | N (%) | N (%) | sig | N (%) | N (%) | sig | N (%) | N (%) | sig |
| **How many times did you use an antibiotic in the last twelve months** | | | | | | | | | | | | | | | |
| **X2** |  |  | <0.001 |  |  | <0.001 |  |  | <0.001 |  |  | <0.001 |  |  | <0.001 |
| **1** | 190 (32.1) | 242 (55.5) | * | 96 (32.4) | 158 (52.5) | * | 94 (32.0) | 84 (62.2) | * | 106 (29.9) | 158 (53.4) | * | 81 (35.1) | 84 (60.0) | * |
| **2** | 196 (33.2) | 106 (24.3) | * | 106 (35.8) | 79 (26.2) | * | 89 (30.3) | 27 (20.0) | * | 121 (34.2) | 79 (26.7) | * | 74 (32.0) | 27 (19.3) | * |
| **>=3** | 205 (34.7) | 88 (20.2) | * | 94 (31.8) | 64 (21.3) | * | 111 (37.8) | 24 (17.8) | * | 127 (35.9) | 59 (19.9) | * | 76 (32.9) | 29 (20.7) | * |
| **It is good to keep remaining antibiotic doses at home for later use upon need** | | | | | | | | | | | | | | | |
| **X2** |  |  | **<0.001** |  |  | **0.193** |  |  | **0.086** |  |  | **0.002** |  |  | **0.025** |
| Agree | 251 (35.1) | 101(23.2) | * | 79 (22.32) | 62 (20.6) | NS | 84 (23.9) | 39 (28.9) | NS | 139 (32.6) | 62 (20.9) | * | 107 (39.5) | 39 (27.9) | * |
| Do not agree | 406 (57.4) | 303(69.5) | * | 264 (74.58) | 221 (73.4) | NS | 247 (70.2) | 82(60.7) | * | 263 (61.6) | 211 (71.3) | * | 140 (51.7) | 92 (65.7) | * |
| Do not know | 50 (7.1) | 32(7.3) | NS | 11(3.11) | 18 (6.0) | NS | 21(6.0) | 14(10.4) | NS | 25 (5.9) | 23 (7.8) | NS | 24 (8.9) | 9 (6.4) | NS |
| **I don’t mind using antibiotics from a friend or a relative without consulting a doctor** | | | | | | | | | | | | | | | |
| **X2** |  |  | 0.117 |  |  | 0.196 |  |  | 0.943 |  |  | .509 |  |  | 0.251 |
| Agree | 163(23.1) | 87 (20) | NS | 79 (22.3) | 55 (18.3) | NS | 84 (23.9) | 32 (23.7) | NS | 98 (23.0) | 60 (20.3) | NS | 63 (23.2) | 27 (19.3) | NS |
| Do not agree | 512(72.4) | 337 (77.3) | NS | 264 (74.6) | 241 (80.1) | NS | 247 (70.2) | 96 (71.1) | NS | 315 (73.8) | 229 (77.4) | NS | 190 (70.1) | 108 (77.1) | NS |
| Do not know | 32(4.5) | 12 (2.8) | NS | 11 (3.1) | 5 (1.7) | NS | 21 (6.0) | 7 (5.2) | NS | 14 (3.3) | 7 (2.4) | NS | 18 (6.6) | 5 (3.6) | NS |
| **I don’t mind buying an antibiotic from a pharmacy without a prescription** | | | | | | | | | | | | | | | |
| **X2** |  |  | 0.53 |  |  | 0.001 |  |  | 0.579 |  |  | 0.789 |  |  | 0.451 |
| Agree | 386 (54.6) | 227 (52.1) | NS | 301 (66.4) | 160 (53.2) | * | 135 (44.4) | 67 (49.6) | NS | 212 (49.6) | 139 (47.0) | NS | 166 (61.3) | 88 (62.9) | NS |
| Do not agree | 299 (42.3) | 198 (45.4) | NS | 141 (31.1) | 134 (44.5) | * | 158 (52.0) | 64 (47.4) | NS | 203 (47.5) | 148 (50.0) | NS | 95 (35.1) | 50 (35.7) | NS |
| Do not know | 22 (3.1) | 11 (2.5) | NS | 11 (2.4) | 7 (2.3) | NS | 11 (3.6) | 4 (3.0) | NS | 12 (2.8) | 9 (3.0) | NS | 10 (3.7) | 2 (1.4) | NS |
| **I know how to use the antibiotic through reading the accompanied leaflet without consulting with a doctor or a pharmacist** | | | | | | | | | | | | | | | |
| **X2** |  |  | 0.314 |  |  | 0.18 |  |  | 0.294 |  |  | 0.83 |  |  | 0.357 |
| Agree | 242 (34.4) | 144 (33.0) | NS | 114 (32.4) | 105 (34.9) | NS | 128 (36.6) | 39 (28.9) | NS | 151 (35.7) | 99 (33.4) | NS | 87 (32.1) | 45 (32.1) | NS |
| Do not agree | 421 (59.9) | 275 (63.1) | NS | 217 (61.6) | 187 (62.1) | NS | 203 (58.0) | 88 (65.2) | NS | 255 (60.3) | 185 (62.5) | NS | 165 (60.9) | 90 (64.3) | NS |
| Do not know | 40 (5.7) | 17 (3.9) | NS | 21 (6.0) | 9 (3.0) | NS | 19 (5.4) | 8 (5.9) | NS | 17 (4.0) | 12 (4.1) | NS | 19 (7.0) | 5 (3.6) | NS |
| **I buy antibiotics even if the doctor is convinced that it is not needed for my case** | | | | | | | | | | | | | | | |
| **X2** |  |  | <0.001 |  |  | 0.016 |  |  | 0.072 |  |  | 0.072 |  |  | <0.001 |
| Agree | 89 (12.7) | 34 (7.8) | * | 32 (9.1) | 21 (7.0) | NS | 57 (16.3) | 13 (9.6) | NS | 44 (10.4) | 29 (9.8) | NS | 44 (16.3) | 5 (3.6) | * |
| Do not agree | 562 (79.9) | 387 (88.8) | * | 294 (83.3) | 271 (90.0) | * | 267 (76.5) | 116 (85.9) | * | 353 (83.3) | 259 (87.5) | NS | 202 (74.8) | 128 (91.4) | * |
| Do not know | 52 (7.4) | 15 (3.4) | * | 27 (7.6) | 9 (3.0) | * | 25 (7.2) | 6 (4.4) | NS | 27 (6.4) | 8 (2.7) | * | 24 (8.9) | 7 (5.0) | NS |
| **You can stop taking your antibiotic when you start feeling better** | | | | | | | | | | | | | | | |
| **X2** |  |  | <0.001 |  |  | <0.001 |  |  | 0.132 |  |  | <0.001 |  |  | <0.001 |
| Agree | 168 (24.0) | 66 (15.1) | * | 78 (22.2) | 38 (12.6) | * | 90 (25.9) | 28 (20.7) | NS | 96 (22.7) | 46 (15.5) | * | 68 (25.3) | 20 (14.3) | * |
| Do not agree | 488 (69.6) | 364 (83.5) | * | 251 (71.3) | 261 (86.7) | * | 236 (67.8) | 103 (76.3) | NS | 306 (72.3) | 248 (83.8) | * | 178 (66.2) | 116 (82.9) | * |
| Do not know | 45 (6.4) | 6 (1.4) | * | 23 (6.5) | 2 (0.7) | * | 22 (6.3) | 4 (3.0) | NS | 21 (5.0) | 2 (0.7) | * | 23 (8.6) | 4 (2.9) | * |
| **Scope of effectiveness** | | | | | | | | | | | | | | | |
| **X2** |  |  | <0.001 |  |  | <0.001 |  |  | <0.001 |  |  | <0.001 |  |  |  |
| Bacteria | 233 (33.4) | 255 (58.5) | * | 129 (37.0) | 177 (58.8) | * | 104 (29.9) | 78 (57.8) | * | 133 (31.6) | 172 (58.1) | * | 98 (36.6) | 83 (59.3) | * |
| Viruses | 146 (20.9) | 35 (8.0) | * | 70 (20.1) | 21 (7.0) | * | 76 (21.8) | 14 (10.4) | * | 83 (19.7) | 27 (9.1) | * | 61 (22.8) | 8 (5.7) | * |
| Both | 247 (35.4) | 116 (26.6) | * | 124 (35.5) | 86 (28.6) | NS | 123 (35.3) | 30 (22.2) | * | 164 (39.0) | 76 (25.7) | * | 78 (29.1) | 40 (28.6) | NS |
| I don’t know | 72 (10.3) | 30 (6.9) | * | 26 (7.4) | 17 (5.6) | NS | 45 (12.9) | 13 (9.6) | NS | 41 (9.7) | 21 (7.1) | NS | 31 (11.6) | 9 (6.4) | NS |
| **Antibiotics accelerate recovery from common cold** | | | | | | | | | | | | | | | |
| **X2** |  |  | <0.001 |  |  | <0.001 |  |  | <0.001 |  |  | <0.001 |  |  | <0.001 |
| Agree | 348 (49.9) | 133 (30.5) | * | 173 (49.9) | 90 (29.9) | * | 175 (50.0) | 43 (31.9) | * | 210 (50.0) | 87 (29.4) | * | 136 (50.6) | 46 (32.9) | * |
| Do not agree | 283 (40.5) | 272 (62.4) | * | 144 (41.5) | 189 (62.8) | * | 138 (39.4) | 83 (61.5) | * | 178 (42.4) | 188 (63.5) | * | 101 (37.5) | 84 (60.0) | * |
| Do not know | 67 ()9.6 | 31 (7.1) | NS | 30 (8.6) | 22 (7.3) | NS | 37 (10.6) | 9 (6.7) | NS | 32 (7.6) | 21 (7.1) | NS | 32 (11.9) | 10 (7.1) | NS |
| **Runny nose accompanied by a colored discharge requires antibiotic therapy** | | | | | | | | | | | | | | | |
| **X2** |  |  | 0.689 |  |  | .907 |  |  | 0.245 |  |  | 0.948 |  |  | 0.267 |
| Agree | 363 (52.5) | 218 (50.0) | NS | 182 (52.8) | 159 (52.8) | NS | 181 (52.3) | 59 (43.7) | NS | 216 (52.4) | 153 (51.7) | NS | 143 (52.6) | 65 (46.4) | NS |
| Do not agree | 169 (24.4) | 115 (26.4) | NS | 82 (23.8) | 75 (24.9) | NS | 86 (24.9) | 40 (29.6) | NS | 99 (24.0) | 70 (23.6) | NS | 67 (24.6) | 45 (32.1) | NS |
| Do not know | 160 (23.1) | 103 (23.6) | NS | 81 (23.5) | 67 (22.3) | NS | 79 (22.8) | 36 (26.7) | NS | 97 (23.5) | 73 (24.7) | NS | 62 (22.8) | 30 (21.4) | NS |
| **Origin of antibiotic resistance** | | | | | | | | | | | | | | | |
| **X2** |  |  | 0.002 |  |  | 0.011 |  |  | 0.312 |  |  | 0.033 |  |  | 0.155 |
| Human | 119 (17.1) | 45 (10.3) | * | 59 (17.0) | 31 (10.3) | * | 59 (16.9) | 14 (10.4) | NS | 72 (17.2) | 32 (10.8) | * | 45 (16.7) | 13 (9.3) | * |
| Microbes | 200 (28.7) | 162 (37.2) | * | 99 (28.5) | 117 (38.9) | * | 101(28.9) | 45 (33.3) | NS | 128 (30.6) | 115 (38.9) | * | 71 (26.3) | 47 (33.6) | NS |
| Both | 264 (37.9) | 156 (35.8) | NS | 138 (39.8) | 107 (35.5) | NS | 126 (36.1) | 49 (36.3) | NS | 155 (37.1) | 102 (34.5) | NS | 105 (38.9) | 54 (38.6) | NS |
| I do not know | 114 (16.4) | 73 (16.7) | NS | 51 (14.7) | 46 (15.3) | NS | 63 (18.1) | 27 (20.0) | NS | 63 (15.1) | 47 (15.9) | NS | 49 (18.1) | 26 (18.6) | NS |
| **Excessive use of antibiotics increases the chance of creating resistance** | | | | | | | | | | | | | | | |
| **X2** |  |  | <0.001 |  |  | <0.001 |  |  | <0.001 |  |  | <0.001 |  |  | <0.001 |
| Agree | 365 (53.3) | 322 (73.9) | * | 182 (53.5) | 225 (74.8) | * | 182 (52.9) | 97 (71.9) | * | 222 (54.3) | 224 (75.7) | * | 138 (51.7) | 98 (70.0) | * |
| Disagree | 234 (34.2) | 97 (22.2) | * | 119 (35.0) | 68 (22.6) | * | 115 (33.4) | 29 (21.5) | * | 137 (33.5) | 64 (21.6) | * | 95 (35.6) | 33 (23.6) | * |
| I do not know | 86 (12.6) | 17 (3.9) | * | 39 (11.5) | 8 (2.7) | * | 47 (13.7) | 9 (6.7) | * | 50 (12.2) | 8 (2.7) | * | 34 (12.7) | 9 (6.4) | * |
| **Antibiotic resistance is a problem in Jordan** | | | | | | | | | | | | | | | |
| **X2** |  |  | <0.001 |  |  | <0.001 |  |  | <0.001 |  |  | <0.001 |  |  | 0.071 |
| Agree | 311 (44.9) | 262 (60.1) | * | 150 (43.6) | 183 (60.8) | * | 160 (46.1) | 79 (58.5) | * | 192 (46.2) | 185 (62.5) | * | 116 (43.4) | 77 (55.0) | * |
| Disagree | 131 (18.9) | 42 (9.6) | * | 67 (19.5) | 27 (9.0) | * | 64 (18.4) | 15 (11.1) | NS | 84 (20.2) | 26 (8.8) | * | 45 (16.9) | 16 (11.4) | NS |
| I do not know | 250 (36.1) | 132 (30.3) | * | 127 (36.9) | 91(30.2) | NS | 123 (35.4) | 41 (30.4) | NS | 140 (33.7) | 85 (28.7) | NS | 106 (39.7) | 47 (33.6) | NS |
| **I ask a doctor to prescribe an antibiotic for me if he does not prescribe one.** | | | | | | | | | | | | | | | |
| **X2** |  |  | <0.001 |  |  | <0.001 |  |  | <0.001 |  |  | <0.001 |  |  | 0.001 |
| Agree | 257 (37.1) | 92 (21.1) | * | 114 (33.1) | 60 (19.9) | * | 143 (41.2) | 32 (23.7) | * | 155 (37.3) | 65 (22.0) | * | 97 (36.3) | 27 (19.3) | * |
| Disagree | 388 (56.1) | 326 (74.8) | * | 208 (60.5) | 227 (75.4) | * | 179 (51.6) | 99 (73.3) | * | 239 (57.5) | 224 (75.7) | * | 146 (54.7) | 102 (72.9) | * |
| I do not know | 47 (6.8) | 18 (4.1) | NS | 22 (6.4) | 14 (4.7) | NS | 25 (7.2) | 4 (3.0) | NS | 22 (5.3) | 7 (2.4) | NS | 24 (9.0) | 11 (7.9) | NS |
| **I Trust the Dr. decision whether he prescribes an antibiotic or not** | | | | | | | | | | | | | | | |
| **X2** |  |  | <0.001 |  |  | 0.008 |  |  | 0.003 |  |  | 0.001 |  |  | 0.014 |
| Agree | 488 (70.1) | 360 (82.6) | * | 250 (72.3) | 248 (82.4) | * | 237 (67.9) | 112 (83.0) | * | 299 (71.4) | 246 (83.1) | * | 184 (68.7) | 114 (81.4) | * |
| Disagree | 163 (23.4) | 64 (14.7) | * | 76 (22.0) | 44 (14.6) | * | 87 (24.9) | 20 (14.8) | * | 98 (23.4) | 42 (14.2) | * | 63 (23.5) | 22 (15.7) | NS |
| I do not know | 45 (6.5) | 12 (2.8) | * | 20 (5.8) | 9 (3.0) | NS | 25 (7.2) | 3 (2.2) | * | 22 (5.3) | 8 (2.7) | NS | 21 (7.8) | 4 (2.9) | * |
| **The Doctor who does not prescribe antibiotic, when the patient believe he should, is an incompetent doctor** | | | | | | | | | | | | | | | |
| **X2** |  |  | <0.001 |  |  | <0.001 |  |  | <0.001 |  |  | <0.001 |  |  | <0.001 |
| Agree | 156 (22.5) | 32 (7.3) | * | 68 (19.8) | 18 (6.0) | * | 88 (25.2) | 14 (10.4) | * | 87 (20.8) | 18 (6.1) | * | 65 (24.4) | 14 (10.0) | * |
| Disagree | 467 (67.3) | 376 (86.2) | * | 240 (69.8) | 264 (87.7) | * | 226 (64.8) | 112 (83.0) | * | 294 (70.2) | 260 (87.8) | * | 169 (63.5) | 116 (82.9) | * |
| I do not know | 71 (10.2) | 28 (6.4) | * | 36 (10.5) | 19 (6.3) | NS | 35 (10.0) | 9 (6.7) | NS | 38 (9.1) | 18 (6.1) | NS | 32 (12.0) | 10 (7.1) | NS |
| *P values <0.05  NS is not significant  # There were some missing answers in 2010  Correct answer is highlighted | | | | | | | | | | | | | | | |
