# Supplementary material for: Changes in public knowledge and perceptions about antibiotic use and resistance in Jordan: a cross-sectional eight-year comparative study
Source: BMC Public Health. 2021 Apr 19;21:750. doi: 10.1186/s12889-021-10723-x (PMC8054398; doi:10.1186/s12889-021-10723-x)
Supplement: Supplementary file 2 — Additional file 2: Table B. Multinomial logistic regression controlling for gender and insurance as confounders in the surveys case-mix. Odds presented as the odds of the wrong answer relative to the right answer for each possible option. [file 12889_2021_10723_MOESM2_ESM.docx]

Table B

Multinomial logistic regression to control for gender and insurance as confounders in the surveys case-mix.

Odds presented as the wrong answer's odds relative to the correct answer for each possible option*.*

| **Question** | **Answer** | **2010** | | **2018** | |
| --- | --- | --- | --- | --- | --- |
|  |  | **Odds (95% CI)** | **P value** | **Odds (95% CI)** | **P value** |
| How many times did you use an antibiotic in the last twelve months | | | | | |
| Gender  (males relative to females) | 3 times or more vs. one time | 1.159 (0.775-1.734) | 0.473 | 0.707 (0.412-1.212) | 0.207 |
| Gender  (males relative to females) | 2 times vs. one time | 0.827 (0.550-1.243) | 0.361 | 0.652 (0.390-1.088) | 0.102 |
| Insurance  (insured vs. uninsured) | 3 times or more vs. one time | 1.276 (0.843-1.931) | 0.250 | 1.070 (0.637-1.797) | 0.799 |
| Insurance  (insured vs. uninsured) | 2 times vs. one time | 1.248 (0.828-1.882) | 0.289 | 1.534 (0.919-2.562) | 0.102 |
| It is good to keep left out antibiotics for later use | | | | | |
| Gender  (males relative to females) | Agree vs. Do not agree | 1.425(1.031-1.969) | **0.032*** | 1.675 (1.041-2.694) | **0.034*** |
| Gender  (males relative to females) | I do not know vs. Do not agree | 1.197(0.655-2.186) | 0.558 | 2.105 (1.001-4.428) | 0.050 |
| Insurance  (insured vs. uninsured) | Agree vs. Do not agree | 0.657 (0.472-0.915) | **0.013*** | 0.705 (.440-1.131) | 0.148 |
| Insurance  (insured vs. uninsured) | I do not know vs. Do not agree | 0.541 (0.296-0.991) | **0.047*** | 1.144 (.508-2.578) | 0.746 |
| I don't mind using antibiotics from a friend or a relative without consulting a doctor | | | | | |
| Gender  (males relative to females) | Agree vs. Do not agree | 1.137 (0.794-1.628) | 0.484 | 1.464 (0.891-2.404) | 0.132 |
| Gender  (males relative to females) | I do not know vs. Do not agree | 2.362 (1.103-5.061) | **0.027*** | 3.465 (1.072-11.199) | **0.038*** |
| Insurance  (insured vs. uninsured) | Agree vs. Do not agree | 0.923 (0.638-1.334) | 0.669 | 1.063 (0.638-1.771) | 0.815 |
| Insurance  (insured vs. uninsured) | I do not know vs. Do not agree | 0.414 (0.199-0.861) | **0.018*** | 0.694 (0.214-2.254) | 0.543 |
| I don't mind buying an antibiotic from a pharmacy without a prescription | | | | | |
| Gender  (males relative to females) | Agree vs. Do not agree | 0.849 (0.623-1.156) | 0.298 | 0.852 (0.561-1.293) | 0.452 |
| Gender  (males relative to females) | I do not know vs. Do not agree | 0.969 (0.404-2.327) | 0.944 | 1.215 (0.343-4.306) | 0.763 |
| Insurance  (insured vs. uninsured) | Agree vs. Do not agree | 0.609 (0.442-0.839) | **0.002*** | 0.530 (0.349-0.805) | **0.003*** |
| Insurance  (insured vs. uninsured) | I do not know vs. Do not agree | 0.564 (0.233-1.363) | 0.204 | 1.534 (0.320-7.351) | 0.592 |
| I know how to use the antibiotic through reading the accompanied leaflet without consulting with a doctor or a pharmacist | | | | | |
| Gender  (males relative to females) | Agree vs. Do not agree | 1.173 (0.850-1.618) | 0.332 | 0.791 (0.506-1.237) | 0.304 |
| Gender  (males relative to females) | I do not know vs. Do not agree | 0.927 (0.464-1.854) | 0.831 | 1.902 (0.709-5.102) | 0.201 |
| Insurance  (insured vs. uninsured) | Agree vs. Do not agree | 1.102 (0.790-1.536) | 0.567 | 1.061 (0.687-1.637) | 0.790 |
| Insurance  (insured vs. uninsured) | I do not know vs. Do not agree | 0.588 (0.295-1.172) | 0.131 | 1.200 (0.409-3.521) | 0.740 |
| I buy antibiotics even if the doctor is convinced that it is not needed for my case | | | | | |
| Gender  (males relative to females) | Agree vs. Do not agree | 2.188 (1.360-3.518) | **0.001*** | 1.501 (0.723-3.115) | 0.276 |
| Gender  (males relative to females) | I do not know vs. Do not agree | 1.160 (0.648-2.078) | 0.617 | 1.518 (0.527-4.374) | 0.440 |
| Insurance  (insured vs. uninsured) | Agree vs. Do not agree | 0.507 (0.319-0.805) | **0.004*** | 2.918 (1.102-7.729) | **0.031*** |
| Insurance  (insured vs. uninsured) | I do not know vs. Do not agree | 0.630 (0.351-1.131) | 0.122 | 0.575 (0.204-1.625) | 0.297 |
| You can stop taking your antibiotic when you start feeling better | | | | | |
| Gender  (males relative to females) | Agree vs. Do not agree | 1.210 (0.845-1.732) | 0.297 | 1.873 (1.092-3.211) | **0.023*** |
| Gender  (males relative to females) | I do not know vs. Do not agree | 1.065 (0.569-1.993) | 0.844 | 4.817 (0.863-26.882) | 0.073 |
| Insurance  (insured vs. uninsured) | Agree vs. Do not agree | 0.801 (0.556-1.155) | 0.235 | 1.099 (0.620-1.950) | 0.746 |
| Insurance  (insured vs. uninsured) | I do not know vs. Do not agree | 0.528 (0.282-0.988) | **0.046*** | 0.247 (0.044-1.382) | 0.112 |
| Scope of effectiveness – Antibiotics are active against: | | | | | |
| Gender  (males relative to females) | Viruses vs. bacteria | 1.321 (0.866-2.013) | 0.196 | 1.545 (0.745-3.203) | 0.242 |
| Gender  (males relative to females) | Both vs. bacteria | 1.137 (0.788-1.640) | 0.493 | 0.788 (0.481-1.292) | 0.345 |
| Gender  (males relative to females) | I do not know vs. bacteria | 2.201 (1.265-3.830) | **0.005*** | 1.747 (0.808-3.775) | 0.156 |
| Insurance  (insured vs. uninsured) | Viruses vs. bacteria | 0.963 (0.629-1.475) | 0.864 | 1.663 (0.723-3.826) | 0.232 |
| Insurance  (insured vs. uninsured) | Both vs. bacteria | 1.521 (1.041-2.222) | **0.030*** | 0.908 (0.570-1.445) | 0.683 |
| Insurance  (insured vs. uninsured) | I do not know vs. bacteria | 0.850 (0.493-1.467) | 0.559 | 1.157 (0.506-2.643) | 0.730 |
| Antibiotics accelerate recovery from common cold | | | | | |
| Gender  (males relative to females) | Agree vs. Do not agree | 1.079 (0.784-1.485) | 0.639 | 1.081 (0.692-1.689) | 0.732 |
| Gender  (males relative to females) | I do not know vs. Do not agree | 1.393 (0.801-2.423) | 0.240 | 0.848 (0.546-1.317) | 0.462 |
| Insurance  (insured vs. uninsured) | Agree vs. Do not agree | 0.871 (0.627-.211) | 0.413 | 0.929 (0.410-2.105) | 0.860 |
| Insurance  (insured vs. uninsured) | I do not know vs. Do not agree | 0.544 (0.312-0.947) | **0.031*** | 0.936 (0.422-2.075) | 0.870 |
| Runny nose accompanied by a colored discharge requires antibiotic therapy | | | | | |
| Gender  (males relative to females) | Agree vs. Do not agree | 0.932 (0.642-1.353) | 0.711 | 0.706 (0.433-1.150) | 0.162 |
| Gender  (males relative to females) | I do not know vs. Do not agree | 0.918 (0.591-1.426) | 0.702 | 1.495 (0.930-2.404) | 0.097 |
| Insurance  (insured vs. uninsured) | Agree vs. Do not agree | 1.044 (0.714-1.527) | 0.825 | 1.024 (0.585-1.793) | 0.934 |
| Insurance  (insured vs. uninsured) | I do not know vs. Do not agree | 1.084 (0.691-1.700) | 0.726 | 1.566 (0.888-2.760) | 0.121 |
| Origin of antibiotic resistance | | | | | |
| Gender  (males relative to females) | Humans vs. microbes | 1.008 (0.635-1.601) | 0.972 | 1.175 (0.572-2.411) | 0.661 |
| Gender  (males relative to females) | Both vs. microbes | 0.897 (0.618-1.304) | 0.570 | 1.180 (0.728-1.913) | 0.502 |
| Gender  (males relative to females) | I do not t know vs. microbes | 1.293 (0.808-2.070) | 0.284 | 1.510 (0.839-2.718) | 0.169 |
| Insurance  (insured vs. uninsured) | Humans vs. microbes | 0.874 (0.542-1.409) | 0.581 | 1.012 (0.488-2.097) | 0.975 |
| Insurance  (insured vs. uninsured) | Both vs. microbes | 0.832 (0.566-1.223) | 0.349 | 0.776 (0.483-1.247) | 0.295 |
| Insurance  (insured vs. uninsured) | I do not t know vs. microbes | 0.687 (0.426-1.108) | 0.124 | 0.750 (0.416-1.351) | 0.338 |
| Excessive use of antibiotics increases the chance of creating resistance | | | | | |
| Gender  (males relative to females) | Do not agree vs. Agree | 0.968 (0.693-1.352) | 0.849 | 0.984 (0.599-1.616) | 0.950 |
| Gender  (males relative to females) | I do not know vs. Agree | 1.178 (0.728-1.906) | 0.505 | 2.530 (0.943-6.786) | 0.065 |
| Insurance  (insured vs. uninsured) | Do not agree vs. Agree | 0.905 (0.644-1.272) | 0.564 | 0.848 (0.523-1.374) | 0.503 |
| Insurance  (insured vs. uninsured) | I do not know vs. Agree | 0.897 (0.549-1.464) | 0.663 | 0.401 (0.150-1.076) | 0.070 |
| Antibiotic resistance is a problem in Jordan | | | | | |
| Gender  (males relative to females) | Do not agree vs. Agree | 0.883 (0.583-1.338) | 0.559 | 1.268 (0.639-2.518) | 0.497 |
| Gender  (males relative to females) | I do not know vs. Agree | 0.909 (0.647-1.276) | 0.581 | 1.033 (0.655-1.627) | 0.890 |
| Insurance  (insured vs. uninsured) | Do not agree vs. Agree | 1.154 (0.748-1.780) | 0.518 | 0.682 (0.346-1.344) | 0.269 |
| Insurance  (insured vs. uninsured) | I do not know vs. Agree | 0.813 (0.576-1.148) | 0.240 | 0.754 (0.483-1.176) | 0.213 |
| I ask a doctor to prescribe an antibiotic for me if he does not prescribe one | | | | | |
| Gender  (males relative to females) | Agree vs. Do not agree | 1.454 (1.053-2.009) | **0.023*** | 1.228 (0.752-2.006) | 0.411 |
| Gender  (males relative to females) | I do not know vs. Do not agree | 1.401 (0.752-2.608) | 0.288 | 0.615 (0.196-1.932) | 0.405 |
| Insurance  (insured vs. uninsured) | Agree vs. Do not agree | 0.926 (0.665-1.291) | 0.651 | 1.107 (0.667-1.838) | 0.695 |
| Insurance  (insured vs. uninsured) | I do not know vs. Do not agree | 0.534 (0.287-0.995) | **0.048*** | 0.284 (0.107-0.756) | **0.012*** |
| I trust the Dr. decision whether he prescribes an antibiotic or not | | | | | |
| Gender  (males relative to females) | Do not agree vs. Agree | 1.248 (0.870-1.792) | 0.229 | 1.002 (0.564-1.779) | 0.996 |
| Gender  (males relative to females) | I do not know vs. Agree | 1.461 (0.774-2.761) | 0.242 | 0.736 (0.195-2.771) | 0.650 |
| Insurance  (insured vs. uninsured) | Do not agree vs. Agree | 0.930 (0.642-1.346) | 0.699 | 0.885 (0.504-1.552) | 0.669 |
| Insurance  (insured vs. uninsured) | I do not know vs. Agree | 0.612 (0.325-1.152) | 0.128 | 0.917 (0.270-3.110) | 0.889 |
| The Doctor who does not prescribe antibiotic, when the patient believe he should, is an incompetent doctor | | | | | |
| Gender  (males relative to females) | Agree vs. Do not agree | 1.476 (1.015-2.146) | **0.042*** | 1.799 (0.863-3.751) | 0.117 |
| Gender  (males relative to females) | I do not know vs. Do not agree | 1.081 (0.650-1.800) | 0.764 | 1.108 (0.486-2.527) | 0.807 |
| Insurance  (insured vs. uninsured) | Agree vs. Do not agree | 0.728 (0.498-1.063) | 0.100 | 0.586 (0.281-1.221) | 0.153 |
| Insurance  (insured vs. uninsured) | I do not know vs. Do not agree | 0.677 (0.405-1.130) | 0.136 | 0.806 (0.361-1.800) | 0.599 |
