# Supplementary material for: Changes in public knowledge and perceptions about antibiotic use and resistance in Jordan: a cross-sectional eight-year comparative study
Source: BMC Public Health. 2021 Apr 19;21:750. doi: 10.1186/s12889-021-10723-x (PMC8054398; doi:10.1186/s12889-021-10723-x)
Supplement: Supplementary file 3 — Additional file 3. Questionnaire in English version. [file 12889_2021_10723_MOESM3_ESM.docx]

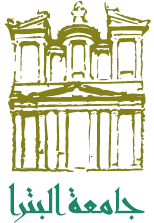
**كلية الصيدلة والعلوم الطبية**

**Faculty of Pharmacy and Medical Sciences**

**University of Petra**

**جامعة البترا**

**Research Title:**

**Public awareness of the problem of antibiotic resistance and its causes in Jordan.**

**Search goal:
This research aims to assess public awareness on the problem of antibiotic resistance and the causes of this problem in Jordan. We would like your cooperation by filling out this questionnaire with accuracy. The information will be treated with complete confidentiality and will be used for scientific research purposes only.**

**Demographics**

**Gender; male female**

**Age**

**Medical Insurance yes no**

***Antibiotic use:***

**7. Do you know the name of one or two medical products that are considered antibiotics?**

A Yes  B No

**8. If your answer to the previous question (7) Yes, how many times have you used antibiotics in the previous 12 months?**

A. once  B twice.  C three times or more.

**9. It is good to keep the remaining dose of antibiotics at home so that they can be used when needed later.**

A agree with this phrase.  B do not agree with this phrase .  C I don't know.

**10. I don't mind getting antibiotics from a friend or relative without referring to a doctor.**

A. agree with this phrase. B. do not agree with this phrase. C. I don't know.

**11. I don't mind getting antibiotics by buying them from a pharmacist without a prescription.**

A. agree with this phrase.  B. do not agree with this phrase. C. I don't know.

**12. I Learn how to use antibiotics from the medical dispatch attached to the drug without asking a doctor or pharmacist.**

A. agree with this phrase. B do not agree with this phrase. C. I don't know.

**13. I buy antibiotics, although the doctor believes they are useless in my case.**

A. agree with this phrase. B. do not agree with this phrase. C. I don't know.

**14. If you feel better after taking half of your antibiotic dose, you can stop taking it.**

A agree with this phrase. B do not agree with this phrase. C I don't know.

***Knowledge of the effect of antibiotics and the causes of resistance:***

**15. Antibiotics are effective against:**

A.  B viruses. C. the two together. D. I don't know.

**16. Antibiotics can be used in the following cases: (you can indicate more than one answer if you like)**

1. leaching (increased nasal discharge/cough). F
2. Feeling pain in the throat. G cough for more than a week.
3. bronchitis. H having heat associated with inflammation.
4. urinary tract infection. I feel pain in the teeth/gums.
5. I don't know.

**17. Antibiotics speed up the recovery process from colds/ colds.**

A. agree with this phrase. B. do not agree with this phrase. C. I don't know.

**18. Leaching/colds accompanied by mucus of color that needs an antibiotic to recover from.**

A. agree with this phrase. B. do not agree with this phrase. C. I don't know.

**19. Antibiotic resistance is created by:**

A. man  B.  germs. C.  the two together. D.  I don't know.

**20.**Excessive use of **antibiotics increases the chances of resistance to them.**

A. agree with this phrase. B. do not agree with this phrase. C. I don't know.

**21. Antibiotic resistance can: (you can indicate more than one answer if you like)**

1. increases the duration of the disease.
2. leads to hospitalization.
3. increases the number of visits to the doctor.
4. increases the need for more expensive medications with more side effects.
5. I don't know.

**22. Antibiotic resistance is a problem in Jordan.**

A. agree with this phrase. B. do not agree with this phrase. C. I don't know.

***The patient's relationship with the doctor/pharmacist with regard to the use of antibiotics:***

**23. Ask your doctor to prescribe an antibiotic if he does not.**

A agree with this phrase. B do not agree with this phrase. C I don't know.

**24. I trust the doctor's decision whether or not he prescribes an antibiotic.**

A agree with this phrase. B do not agree with this phrase. C I don't know.

**25. A doctor who does not dispense antibiotics to the patient (although the patient believes he or she needs an antibiotic) is not good.**

A agree with this phrase. B do not agree with this phrase. C, I don't know.

.
